# A Triple Culture Model of the Blood-Brain Barrier Using Porcine Brain Endothelial cells, Astrocytes and Pericytes

**DOI:** 10.1371/journal.pone.0134765

**Published:** 2015-08-04

**Authors:** Louiza Bohn Thomsen, Annette Burkhart, Torben Moos

**Affiliations:** Laboratory of Neurobiology, Department of Health Science and Technology, Aalborg University, Aalborg, Denmark; Hungarian Academy of Sciences, HUNGARY

## Abstract

*In vitro* blood-brain barrier (BBB) models based on primary brain endothelial cells (BECs) cultured as monoculture or in co-culture with primary astrocytes and pericytes are useful for studying many properties of the BBB. The BECs retain their expression of tight junction proteins and efflux transporters leading to high trans-endothelial electric resistance (TEER) and low passive paracellular permeability. The BECs, astrocytes and pericytes are often isolated from small rodents. Larger species as cows and pigs however, reveal a higher yield, are readily available and have a closer resemblance to humans, which make them favorable high-throughput sources for cellular isolation. The aim of the present study has been to determine if the preferable combination of purely porcine cells isolated from the 6 months old domestic pigs, i.e. porcine brain endothelial cells (PBECs) in co-culture with porcine astrocytes and pericytes, would compare with PBECs co-cultured with astrocytes and pericytes isolated from newborn rats with respect to TEER value and low passive permeability. The astrocytes and pericytes were grown both as contact and non-contact co-cultures as well as in triple culture to examine their effects on the PBECs for barrier formation as revealed by TEER, passive permeability, and expression patterns of tight junction proteins, efflux transporters and the transferrin receptor. This syngenic porcine *in vitro* BBB model is comparable to triple cultures using PBECs, rat astrocytes and rat pericytes with respect to TEER formation, low passive permeability, and expression of hallmark proteins signifying the brain endothelium (tight junction proteins claudin 5 and occludin, the efflux transporters P-glycoprotein (PgP) and breast cancer related protein (BCRP), and the transferrin receptor).

## Introduction

Brain endothelial cells (BECs) denote the blood-brain barrier (BBB) and form a major physical restraint on the transport into the brain of several molecules present in blood plasma for transport. The BECs are non-fenestrated, rich in mitochondria, high in concentrations of drug- and nutrient metabolizing enzymes, but low in vesicles involved in endocytotic and transcytotic activity [1,2]. The BECs are also closely connected with intermingling tight junctions and adherence junctions[3–6]. Pericytes and end-feet of astrocytes form close contact with the BECs and participate in the formation, regulation and maintenance of the integrity of the BBB[1,5,7–11].

Modeling the BBB has been an important issue for decades. Experimental conditions are often more controllable *in vitro* than *in vivo*, and they are overall also more ethically acceptable due to the reduced number of animals applied per study when performed *in vitro*. The BBB *in vitro* forms many characteristics of the *in vivo* conditions, e.g. robust tight junction expression and luminal to abluminal molecular transport indicative of defined polarity. Both primary and immortalized cells are being used for *in vitro* studies of the BBB. Primary BECs have been isolated and cultured from most mammals with the foremost originating from rats, mice, pigs, cows, and even humans (e.g.[12–15]).

The mechanisms that induce polarization of the BECs are not fully understood, but astrocytes are known to secrete a number of substances that participates in the induction of the BBB, e.g. basic fibroblast growth factor (bFGF) and angiopoietin 1 (ANG1)[5,7]. Co-culturing with astrocytes is often required when BBB models are used for studying transcellular transport[10,16,17]. Pericytes are also known to induce a tighter BBB [7,11,18], which suggests that both astrocytes and pericytes could be cultured with BECs as triple cultures to obtain optimized conditions. Furthermore, raising intracellular cAMP in BECs strengthens the BBB properties and is useful when establishing an *in vitro* BBB model [13,19,20].

Primary BECs are often isolated from small mammals. The ready availability and higher cellular yield, however, also make larger species like pigs and cow’s favorable high-throughput sources for BECs, astrocytes and pericytes. An *in vitro* BBB model based on porcine brain endothelial cells (PBECs) has several advantages when compared to those of *in vitro* rodent BBB models: i) higher cellular yield per animal, ii) PBECs retain many of the important BBB features, iii) human and porcine genome, anatomy, physiology, and disease progression are comparable, iv) porcine brains are by-products from the abattoir and therefore inexpensive, v) their usage for research is more ethically acceptable [21]. Recently, porcine disease models were established for studying human diseases including transgenic pig models, which make the PBECs an even stronger competitor to the rodent models [21]. In the past, PBECs were mainly cultured as monoculture or in co-culture with primary rat astrocytes isolated from neonatal rats or rat astrocytes cell lines e.g. C6 glioma cells (e.g.[14,17,21–24]). Such co-cultures were, therefore, constructed from two different species, which could influence the barrier function and gene expression of the PBECs. Rat astrocytes are often derived from pups due to their ability to grow faster than astrocytes obtained from older animals [25]. Deriving cells from laboratory animals is very expensive and requires large amounts of animal sacrifices only for this purpose.

In the present study, PBECs, astrocytes and pericytes were isolated from 6 months old domestic pig brains donated and considered a waste product by the local abattoir. The aim of the present study was to establish a triple culture based entirely on porcine cells i.e. PBECs, astrocytes and pericytes and to determine if this preferable cellular combination for BBB formation would compare to PBECs co-cultured with rat astrocytes and pericytes isolated from newborn rat pups. Porcine or rat astrocytes and pericytes were cultured in both contact and non-contact co-culture with PBECs to examine their effects on the PBECs for barrier formation as revealed by formation of trans-endothelial electric resistance (TEER), loss of passive permeability, and expression patterns of BEC specific proteins. The results show that primary porcine astrocytes and pericytes are useable for triple culture with PBECs instead of primary rat astrocytes and pericytes, as equally high TEER values, low passive permeability and expression of hallmarks of BECs can be observed.

## Materials and Methods

### Cell culture

The animal work was conducted according to Danish and European regulations. All rats were obtained from the Animal Facility at Aalborg University Hospital. The animals were fed and housed under a 12/12 h dark/light cycle and had free access to food and water until they were euthanized. Animal handling was done by researchers who have passed a FELASA category A or C Laboratory Animal Science course issued by the Danish Experimental Animal Inspectorate. No experimental permission or ethical approvals are necessary when the animals are not used for experiments but instead are immediately euthanized according to Danish and European legislation. The pig brains were obtained from the local abattoir (Danish Crown, DK), which are obligated to follow the Danish regulations within animal welfare and are under constant supervision by the Danish and European Food Standard Agency

PBECs were derived from 6 months old domestic pig brains. The brains were collected and transported on ice to the Laboratory of Neurobiology, Aalborg University, Denmark. The isolation of the PBECs was started within 2–3 hours from termination of the animal. The PBECs were isolated using a slightly modified protocol previously described [18][26]. Meninges were removed and approximately 12–15 g cortex, containing as little white matter as possible (approximately 20%), were collected in DMEM-F12 (Life Technology, Naerum, Denmark, DK) and cut into small pieces using scalpels. The tissue was digested in collagenase II (Life Technology) and DNase I (Roche, Hvidovre, Denmark, DK) for 75 min at 37°C, and purified in 20% BSA, followed by a second enzyme treatment with collagenase/dispase (Roche) and DNase I for 50 min at 37°C. Microvessels were collected using a 33% Percoll gradient (Sigma-Aldrich, Brondby, Denmark, DK). The isolated microvessel fragments were finally plated on to 60 mm^2^ plastic dishes coated with collagen IV (Sigma-Aldrich) and fibronectin (Sigma-Aldrich). PBECs were maintained in DMEM/F12 supplemented with 10% plasma-derived serum (First Link, Wolverhampton, United Kingdom, UK), basic fibroblast growth factor (Roche), heparin (Sigma-Aldrich), insulin, transferrin, sodium selenite (Roche) and gentamicin sulphate (10μg/ml) and cultured in an incubator with humidified 5% CO _2_ / 95% air at 37°C. Puromycin (Sigma-Aldrich) was added to the media for the first 3 days to obtain a pure culture of PBECs. After 3 days the cells were passaged and seeded on to 1.12 cm^2^ Millicell hanging culture inserts with 1μm pore size (Millipore) in a density of 100.000 cells per insert. All experiments on PBECS were conducted on passage one.

Cerebral porcine pericytes were obtained by culturing a cell fraction obtained from the PBECs isolation protocol. When the microvessels were collected from the Percoll gradient, the underlying cell fraction in the gradient was collected as pericytes. Pericyte survival and proliferation were favored over PBECs by i) using uncoated dishes, ii) addition of puromycin, and iii) DMEM supplemented with 10% fetal calf serum and gentamicin sulphate. Only passage 1 or 2 of primary porcine pericytes were used in this study.

Mixed cultures of porcine glia cells were additionally obtained from the brain of the 6 months old domestic pigs. Approximately 1.5–2 g of cortical pieces were collected and mechanically dissociated in DMEM supplemented with 10% fetal bovine serum and gentamicin sulphate. Dissociated cells were seeded into culture flasks until they reached confluence, frozen in media supplemented with DMSO and FCS in a -80°C freezer for 24 hours, and then moved to a -140°C freezer until use. It was evidenced by immunocytochemistry that the mixed glial cell cultures consisted mainly of astrocytes and only a few microglial cells, and therefore the mixed glial cell population is referred to below as porcine astrocytes. The porcine astrocytes were thawed two-three weeks before establishment of co-culture models and seeded in 12 well dishes to obtain a confluent layer for co-culture. During the first three days of culturing of the porcine cells, the antibiotic chloramphenicol (Sigma-Aldrich) was added to the culture medium due to the high occurrence of methicillin resistance staphylococcus aureus (MRSA) (CC398) in Danish pigs.

Mixed cultures of rat glial cells were also isolated from neonatal Sprague Dawley rats as previously described by Nakagawa et al and Fazakas et al [18],[26]. The rats were obtained from the Animal Facility at Aalborg University Hospital. The rats were rapidly decapitated by scissor, and their brains removed from the scull. From here on the procedure for isolation of astrocytes and pericytes described above was followed. Pericytes were derived from 2–3 weeks old Sprague Dawley rats as previously described by Nakagawa et al and Fazakas et al [18],[26]. The rats were deeply anesthetized by a subcutaneous injection of 0.5 ml / 10 g body weight of Hypnorm/Dormicum (Fentanyl/Fluanisone mixed with Midazolam and sterile water in a ratio of 1:1:2). The rat heads were rinsed with 70% ethanol and 10% poly (vinylpurrolidone)-iodine complex. The head was separated from the body by scissor. The brains were gently removed from the scull, and the forebrain collected in ice-cold PBS. The meninges and any visible white matter were carefully removed. From here on the protocol for isolation of porcine pericytes was used.

### *In vitro* BBB model construction

Thirteen different *in vitro* BBB models were constructed using the five different primary cell types, i.e. PBECs, porcine astrocytes, porcine pericytes, rat astrocytes, and rat pericytes (S1 Fig). The thirteen different models were subdivided into four different types of *in vitro* BBB models. The simplest *in vitro* BBB model was a monoculture of PBECs, in which the PBECs were cultured on the upper side of the hanging culture inserts. The second type of *in vitro* BBB model was a non-contact co-culture model in which the culture insert containing PBECs were cultured together with porcine astrocytes, rat astrocytes, porcine pericytes or rat pericytes, which were located on the bottom of the 12 well culture dish. The third type was a contact co-culture models in which porcine astrocytes, rat astrocytes, porcine pericytes or rat pericytes was cultured on the bottom of the culture insert, together with PBECs, which were cultured on the upper side of the culture insert. The fourth and final type of *in vitro* BBB model was a triple culture model. In this model, the PBECs were cultured on the upper side of the culture inserts, while porcine or rat pericytes were cultured on the bottom side of the culture inserts and porcine or rat astrocytes were seeded on the bottom of the culture dish. When the PBECs had reached confluence approximately 24 hours after seeding, the PBECs in all thirteen *in vitro* BBB models were supplied once with 550nM hydrocortisone (Sigma-Aldrich), 250μM cAMP (Sigma-Aldrich) and 17.5μM RO-201724 (Sigma-Aldrich) to further induce BBB characteristics.

When constructing the contact co-cultures the astrocytes or pericytes were seeded in a density of 80.000 cells per insert. The hanging cell culture insert was turned upside down in a large petri dish and coated with poly-l-lysine for seeding astrocytes. The appropriate amount of cells was resuspended in 100μl media per insert and seeded on the insert. The closed petri dish with the hanging cell culture inserts were then placed in an incubator for 3–4 hours until attached. The inserts were then placed hanging into a 12 well culture dish supplied with media in both insert and well and incubated for three days, until PBECs were seeded in the inserts as described previously.

For construction of non-contact co-cultures with pericytes a density of 20.000cells/cm^2^ was seeded into a 12 well culture dish and incubated for 2–3 weeks before co-culture studies was conducted. Astrocytes cultured in non-contact co-cultures were seeded as described previously.

### Evaluation of barrier integrity

The barrier integrity of the different *in vitro* BBB models was evaluated by measurement of TEER and permeability to radiolabeled mannitol (Pelkin Elmer, Skovlunde, Denmark, DK). TEER was measured using a Millicell epithelial-volt-ohm meter and chopstick electrodes (Millipore). The TEER value was calculated as the measured values minus measurements of coated but cell free culture inserts for monoculture and contact co-culture or coated inserts with either astrocytes or pericytes on the bottom of the insert for contact-co-cultures. The difference was multiplied with the area of the culture insert (1.12cm^2^), resulting in a TEER value given as a mean in Ω x cm^2^ ± standard deviation. TEER values were obtained from 35 culture inserts with PBECs cultured in monoculture (n = 35) and from 13–31 culture inserts with PBECs cultured in co-cultures and triple cultures (n = 13–31).

Passive permeability was analyzed by the addition of 1μCi 3H-D-Mannitol (Specific activity 14.2 Ci/mol) to the upper chamber of a culture insert. The passive permeability was performed on three individual culture insert of each of the thirteen different *in vitro* models (13 x n = 3). The culture plate was placed on a rocking table at 37°C for 120 min. Samples of 100 μl were collected from the upper chamber at 0 and 120 min, and from the lower chamber at 0, 15, 30, 60 and 120 min. The samples were replaced with 100 μl fresh culture medium. Samples were added with Ultima Gold liquid Scintillations fluid (Pelkin Elmer) and counted in a liquid scintillations counter. The total amount of millimoles transported in each well was plotted against time. The flux at steady state was calculated as the slope of the straight line divided by the area of the culture insert (1.12 cm^2^). Finally, the apparent permeability (Papp) was calculated by dividing the flux at steady state with the initial concentration in the donor upper compartment. The calculated Paap data were plotted against TEER values for each individual culture insert. Data from TEER and passive permeability were analyzed by the GraphPad Prism 5.0 software using a 1-way ANOVA with Bonferroni’s multiple comparisons test.

### Immunocytochemistry

All primary and secondary antibodies were dissolved in PBS (1:200) prior to labeling. The PBECs, astrocytes and pericytes were fixed in 4% paraformaldehyde and blocked in PBS supplemented with 0.2% Triton-X-100 and 3% bovine serum albumin for 1 hour. The PBECs were stained with polyclonal rabbit anti-claudin-5 (Sigma-Aldrich, cat. no. SAB4502981, lot 310145) and polyclonal rabbit anti-ZO-1 (Invitrogen, cat. no. 617300, lot 1087989A). Mixed glial cells were stained with rabbit anti-glial fibrillary acidic protein (GFAP)(DAKO, DK, cat. no. Z0334, lot 20003791) and Texas Red labelled Lycopersicon Esculentum (Tomato) Lectin (Vector Labs, Peterborough, United Kingdom, cat. no. TL1176, lot W0812). Pericytes were stained with monoclonal mouse anti-α-smooth muscle actin (α-SMA) (Sigma-Aldrich, cat. no. A5228, lot 091M4832), polyclonal rabbit anti-ZO-1 and rabbit anti-platelet-derived growth factor receptor-beta (PDGFR-β) (Santa Cruz, cat.no.Sc-432, lot K1113). For detection, the cells were subsequently stained with goat anti-rabbit Alexa 488 or goat anti-mouse Alexa 585 (Invitrogen) as the secondary antibodies. All cells were counterstained with DAPI. The Millicell membranes were cut out of the inserts and mounted on glass slides in fluorescent mounting media (Dako, Denmark) and cover slips were placed upon the membranes.

### RT-qPCR analysis

All reagents for RT-qPCR were obtained from Thermo Scientific (Slangerup, Denmark, DK), except primers that were purchased from TAG Copenhagen (Frederiksberg, Denmark, DK). RNA was isolated from PBECs from all thirteen *in vitro* BBB model setups and obtained in 3–6 replicates and one replicate consisted of PBECs from 4–6 inserts. RNA was isolated using the GeneJet RNA purification kit. The RNA samples were treated with DNase I to eliminate genomic DNA and 100 ng RNA was converted to cDNA using the RevertAid First Strand cDNA Synthesis Kit. The expression profile of endothelial cell characteristic proteins was assessed with the qPCR technique using primers specific for claudin-5, occludin, transferrin receptor, p-glycoprotein (P-gp) and breast cancer resistance protein (BRCP). Beta actin was used for normalization purpose (Table 1). Each qPCR reaction was performed by mixing 2.5 ng cDNA and 10 pmol of each primer with the Maxima SYBR Green qPCR Mastermix. Each sample was performed in triplicates, while non-reversed RNA and water served as negative controls. The qPCR reactions were 95°C for 10 min, 40 cycles of 95°C for 30 sec, 60°C for 30 sec and 72°C for 30 sec, which were performed using the Stratagene Mx3000P QPCR system (Agilent Technologies, Horsholm, Denmark, DK). The relative expression of mRNA was calculated according to Pfaffl [27] and analysed in the GraphPad Prism 5.0 software using a 1-way ANOVA with Tukey’s multiple comparisons post hoc test.

**Table 1:** The table displays the reference sequence numbers and primer sequences of the six primers used in this study.

Target	Reference sequence	Forward primer	Reverse primer
**Claudin 5**	NM_001161636.1	GTCTTGTCTCCAGCCATGGGTTC	GTCACGATGTTGTGGTCCAGGAAG
**Occludin**	NM_001163647.2	GCCCATCCTGAAGATCAGGTGAC	CTCCACCATATATGTCGTTGCTGGG
**Transferrin receptor**	NM_214001.1	TTGATGATGCTGCTTTCCCTTTCCT	CCATTCTGTTCAACTGAGGAACCCT
**Pgp**	XM_003130205.2	CGATGGATCTTGAAGAAGGCCGAAT	CCAGTTTGAATAGCGAAACATGGCA
**BCRP**	NM_214010.1	GCTATCGAGTGAAAGTGAAGAGTGGCT	AACAACGAAGATTTGCCTCCACCTG
**β-Actin**	XM_003124280.2	CAGAGCGCAAGTACTCCGTGTGGAT	GCAACTAACAGTCCGCCTAGAAGCA

## Results and Discussion

PBECs, astrocytes and pericytes were isolated from 6 months old domestic pigs using a modified version of the protocol originally developed for isolation of rat BECs [18][26]. Thereby, the present *in vitro* BBB model gives the advantage of having all three cell types forming an *in vitro* BBB derived from the same species. The average yield per isolation from 12–15 grams of brain tissues was 8–10 x 10^6^ PBECs and 5.0 x 10^6^ cells for porcine pericytes. On average, 5.0 x 10^7^ porcine astrocytes were isolated from 2 grams of brain tissue. Accordingly, many triple culture experiments are possible using the cells isolated from one single porcine brain. The porcine astrocytes grew slowly in the first week compared to that of the rat astrocytes, but after two weeks of continued culture, no difference in growth pattern was detected between the two astrocyte populations. When grown on collagen/fibronectin coated hanging cell culture inserts, the PBECs acquired the characteristic morphology of brain endothelial cells, seen as a tightly connected, thin monolayer.

### Immunocytochemical stain of PBECs, porcine astrocytes and pericytes

**Fig 1:**
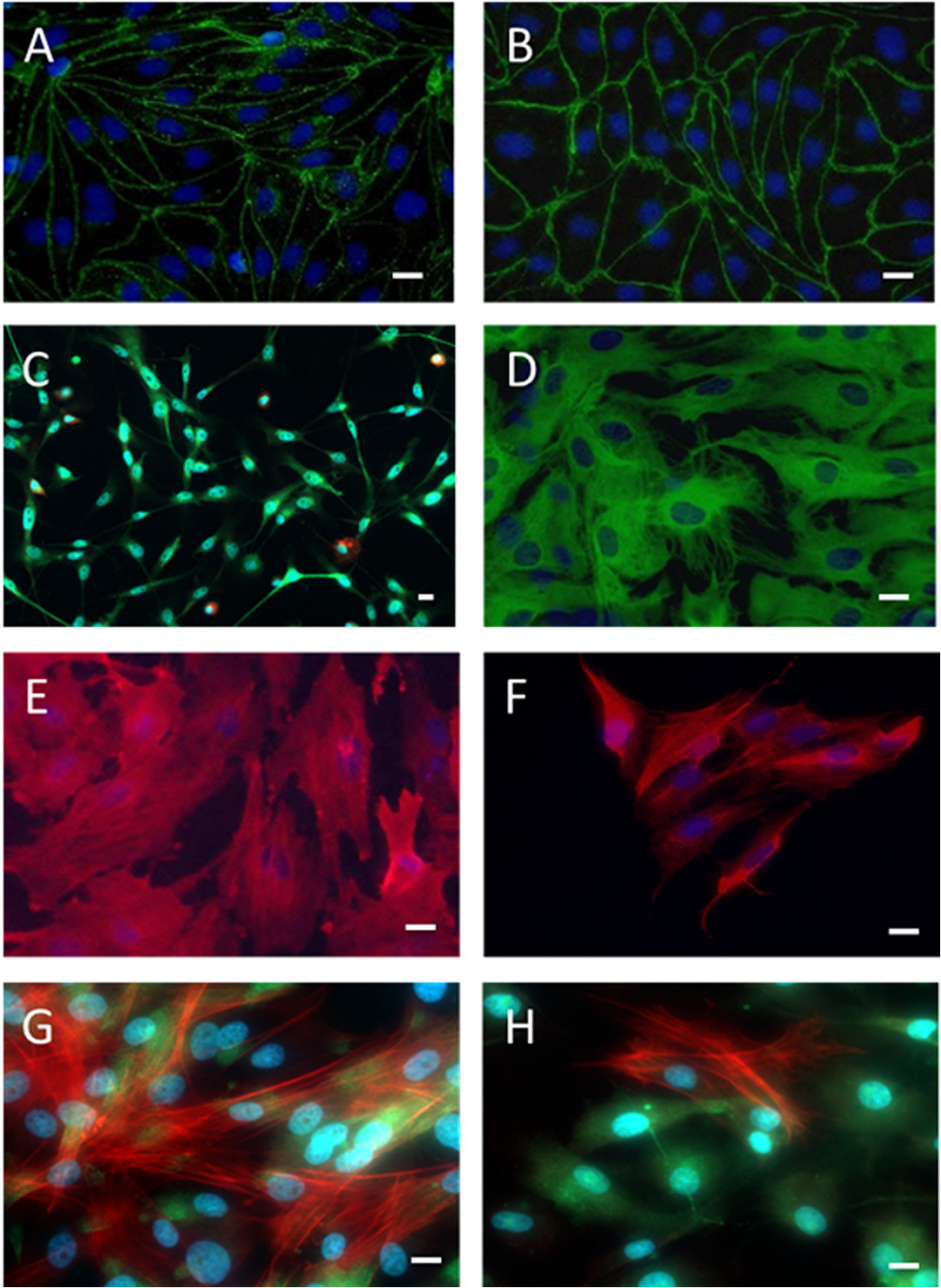
Characterization of primary cell cultures by immunocytochemistry. PBECs express the tight junction proteins Claudin-5 (A, green) and Zonula occludens 1 (B, green) at the cell borders. Porcine mixed glial cells (C) mainly consist of astrocytes which express GFAP (green), and a few (5–10%) microglial cells which express tomato lectin (red). Rat astrocytes (D) express GFAP (green). Porcine pericytes (E) and rat pericytes (F) express alpha-smooth muscle actin (red). Porcine pericytes cultured in monoculture (G) and porcine pericytes cultured in a triple culture with porcine pericytes and PBECs in a triple culture (H) stain for PDGFR-beta (green) and alpha-smooth muscle actin (red). Only a few of the porcine pericytes cultured in triple culture express alpha-smooth muscle actin. Cell nuclei stained with DAPI (blue). Scale bar = 20μm.

Following 5 days of culture in hanging culture inserts, the PBECs expressed claudin-5 and ZO-1 (Fig 1A+1B). Claudin-5 was abundant at cell borders, but also seen in the cytosol. ZO-1 formed a continuous border between the endothelial cells. A possible pericyte contamination in the PBEC culture was reduced with the addition of puromycin to the PBECs for the first three days of culture after isolation [28]. The porcine mixed glial cells mainly consisted of GFAP-positive astrocytes and a few tomato lectin-stained microglia that occurred in the range of 5–10% (Fig 1C). The rat mixed glial cells mainly consisted of GFAP-positive astrocytes (Fig 1D) and a very few microglia (data not shown).

The porcine pericytes stained positive for α-SMA and PDGFR-β, when cultured in monoculture (Fig 1G). Porcine pericytes co-cultured in a triple culture with PBECs and astrocytes also stained positive for PDGFR-β, but only minorities of the pericytes were α-SMA-positive (Fig 1H). These observations are in good accordance with previous studies on differentiating pericytes, which consistently express PDGFR-β irrespective of differential stage, but turn into α-SMA-negative pericytes when subjected to bFGF [29,30]. Furthermore, α-SMA-negative pericytes induce higher TEER than α-SMA-positive pericytes [30]. In the present study, the pericytes were first isolated and cultured as monoculture in bFGF-free media, which resulted in α-SMA-positive pericytes (Fig 1E+1G). When the pericytes were co-cultured with PBECs or cultured as a triple culture with PBECs and astrocytes, bFGF added to the media resulted in α-SMA-negative pericytes (Fig 1H). Rat pericytes were also α-SMA positive in monoculture (Fig 1F), but when cultured in co- or triple cultures fewer were α-SMA positive (data not shown).

### Trans-endothelial electrical resistance of porcine brain endothelial cells

#### TEER monitoring of porcine brain endothelial cells in mono culture

**Fig 2:**
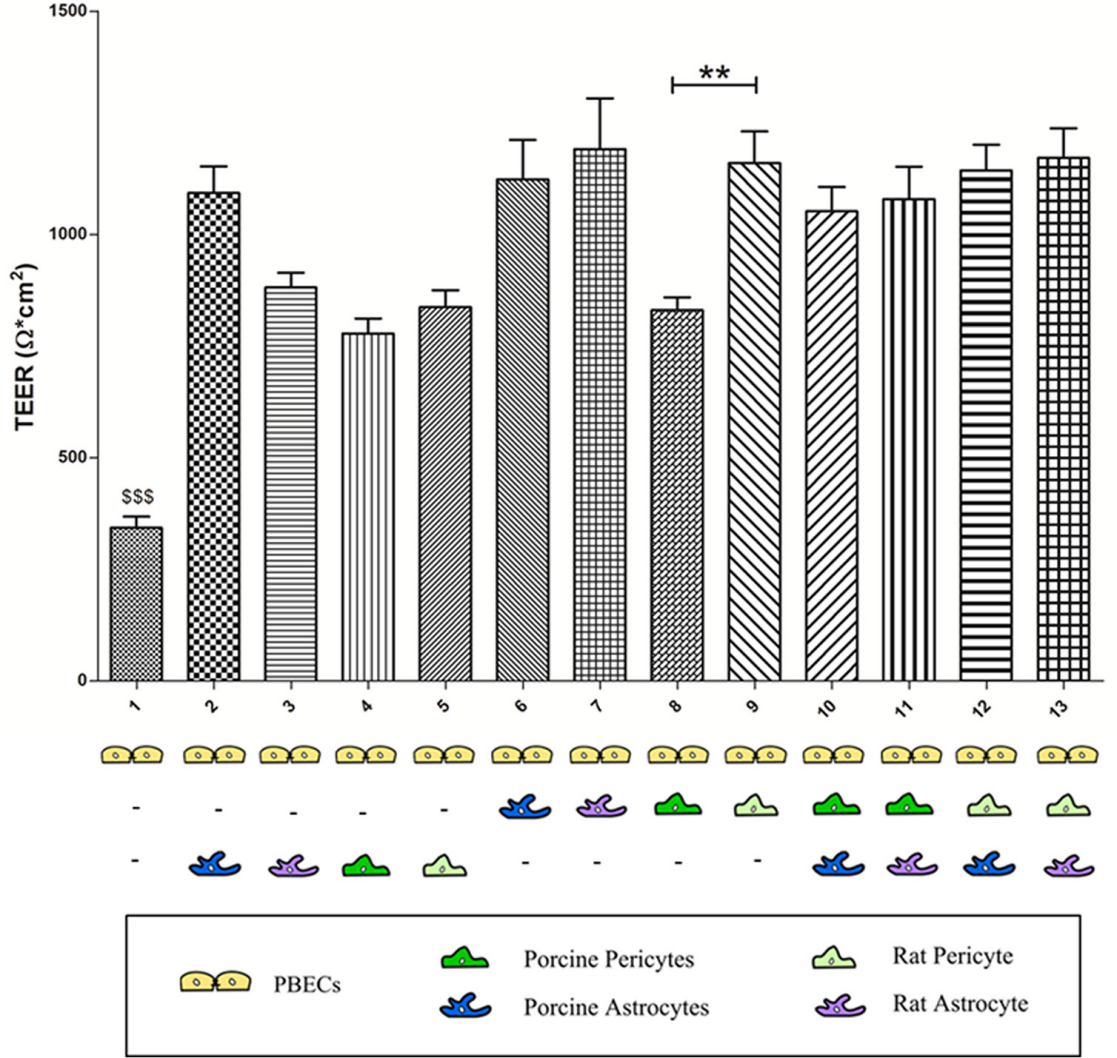
Trans-endothelial electric resistance (TEER) measurement made across PBECs in thirteen co-culture combinations. The mean TEER value of monocultures (n = 35) is significantly lower ($ $ $, P<0.001) than the mean TEER values for all other culture combinations (n = 13–31). Significant difference between the mean TEER value for PBECs cultured in contact co-culture with porcine pericytes (n = 24) compared to PBECs co-cultured in contact co-culture with rat pericytes (n = 23) (**, P<0.01)(n equals number of inserts).

TEER measurements denote a valid real-time monitor of the BBB integrity. Reportedly, primary PBECs form TEER values between 70–1800 Ω x cm^2^ depending on culture conditions [31]. PBECs were previously mainly cultured as pure monocultures or as co-cultures with primary rat astrocytes or astrocytic cell lines like C6 glioma (e.g. [12],[21],[22],[24],[23][31]). We established thirteen different culture combinations with PBECs in co-culture with porcine astrocytes, porcine pericytes, rat astrocytes and/or rat pericytes (S1 Fig). The TEER values monitored as a measure for tightness were on average 344±25 Ω x cm^2^ (n = 35) for the PBECs in mono culture (Fig 2), which is within the range of TEER values found in previous studies on PBECs in monoculture [22,24,32], although it should be noted that a study reports a TEER value of 789±18 Ω x cm^2^ [23]. As expected from previous studies[18,22,32,33], TEER values of the mono cultures were significantly lower than PBECs cultured in co-culture and triple culture (P<0.0001) (Fig 2).

As the pore size and TEER measuring devices affect TEER values, this should be accounted for when comparing studies [31,34]. The TEER values of the present study was measured by chopstick electrodes on PBECs grown on hanging cell culture inserts with a pore size of 1μm. This differs from the before-mentioned studies, which all used a pore size of 0.4μm [22–24,32]. Furthermore two of the before-mentioned studies measured TEER using an Endohm electrode chamber [22,23], whereas the remaining used chopstick electrodes [24,32].

#### TEER monitoring of porcine brain endothelial cells in co-cultures

Co-cultures of PBECs with rat astrocytes beneficially affect TEER values [22,32,33]. Furthermore TEER values in rat BECs can be increased by co-culture with primary rat pericytes [18]. In the present study both rat and porcine astrocytes and pericytes increases the integrity of the PBECs when compared to mono cultures.

The TEER values in PBECs cultured in non-contact co-cultures were 778±33 Ω x cm^2^ (n = 13) with the porcine pericytes, 837±38 Ω x cm^2^ (n = 22) with the rat pericytes, 881±33 Ω x cm^2^ (n = 18) with the rat astrocytes and 1093±60 Ω x cm^2^ (n = 19) with the porcine astrocytes. There were no significant differences between the TEER values obtained on PBECs cultured in non-contact co-cultures when comparing the inductive properties of rat and porcine astrocytes or pericytes. Though, the TEER values for all non-contact cultures indicate that porcine astrocytes are preferable for non-contact co-cultures with PBECs for obtaining high TEER in non-contact culture (Fig 2). Studies made on PBECs cultured in non-contact co-culture with primary rat astrocytes reportedly lead to TEER values in the range of ~400–800 Ω x cm^2^ [17,21,22,35], which is lower than the mean TEER value of 881±33 Ω x cm^2^ obtained with rat astrocytes in the present study.

For the contact co-cultures TEER of PBECs were 831±29 Ω x cm^2^ (n = 24) with the porcine pericytes, 1123±89 Ω x cm^2^ (n = 21) with the porcine astrocytes, 1160±71 Ω x cm^2^ (n = 23) with rat pericytes, and 1192±113 Ω x cm^2^ (n = 15) in the case of rat astrocytes. Only TEER values in PBECs that had been cultured in contact co-cultures with porcine pericytes were significantly lower (P<0.05) than TEER values obtained from PBECs cultured in contact with rat pericytes (Fig 2). Rat pericytes are therefore better at inducing high TEER in PBECs when cultured in contact co-culture in comparison with porcine pericytes. This is not the case when PBECs are cultured in non-contact co-culture with porcine or rat pericytes, where no significant difference was found.

Rat astrocytes are better at inducing high TEER when co-cultured in contact co-culture [35]. In the present study no significant difference was found between TEER obtained from PBECs cultured in contact co-culture compared to non-contact co-culture with rat astrocytes, although the TEER values were higher in contact co-cultures.

When comparing TEER values, rat and porcine astrocytes do not have higher inductive properties on TEER in PBECs than rat and porcine pericytes (Fig 2). This differs from the findings by Nakagawa et al., where TEER was significantly increased by rat pericytes in contact-co-culture with rat BECs compared to astrocytes in either type of co-culture with rat BECs [18].

In conclusion, co-cultures with both porcine or rat astrocytes or pericytes increase TEER values in PBECs. In non-contact co-cultures porcine astrocytes induce the highest mean TEER values, whereas in contact co-culture rat astrocytes induce the highest mean TEER values. Furthermore, in contact co-culture, rat pericytes can be preferred over porcine pericytes.

#### TEER monitoring of porcine brain endothelial cells in triple cultures

Although expected based on studies on rodent triple cultures [18], the present study is the first to show that TEER increases by triple culture with primary porcine pericytes and porcine astrocytes. The TEER values for PBECs cultured in triple culture were 1052±55 Ω x cm^2^ (n = 31) for the triple porcine culture, 1079±73 Ω x cm^2^ (n = 26) with rat astrocytes and porcine pericytes, 1143±58 Ω x cm^2^ (n = 23) with porcine astrocytes and rat pericytes, and 1171±55 Ω x cm^2^ (n = 24) with rat astrocytes and rat pericytes (Fig 2). The TEER values of the four regimens were non-significant, indicating that rat astrocytes and pericytes cannot be preferred over porcine astrocytes and pericytes for triple culturing. Therefore, the results show that an *in vitro* BBB model established from a triple culture of PBECs, porcine astrocytes and porcine pericytes is just as tight as an *in vitro* model based on co-culture of PBECs with rat astrocytes and pericytes.

There was no significant difference in TEER between the eight co-cultures and the four triple cultures. Therefore based on TEER values alone, triple culture has no advantages over co-culture, but likewise co-culture has no advantages over triple culture.

In conclusion, triple culture significantly increased TEER when compared to mono culture but not when compared to co-cultures. No advantage was found, based on TEER, of triple culture with rat astrocytes and pericytes in comparison with porcine astrocytes and pericytes. Researchers could therefore benefit from using an *in vitro* BBB model based solely on porcine cells when taking costs and ethics into consideration.

### Permeability of porcine brain endothelial cells in mono-, co- and triple cultures

**Fig 3:**
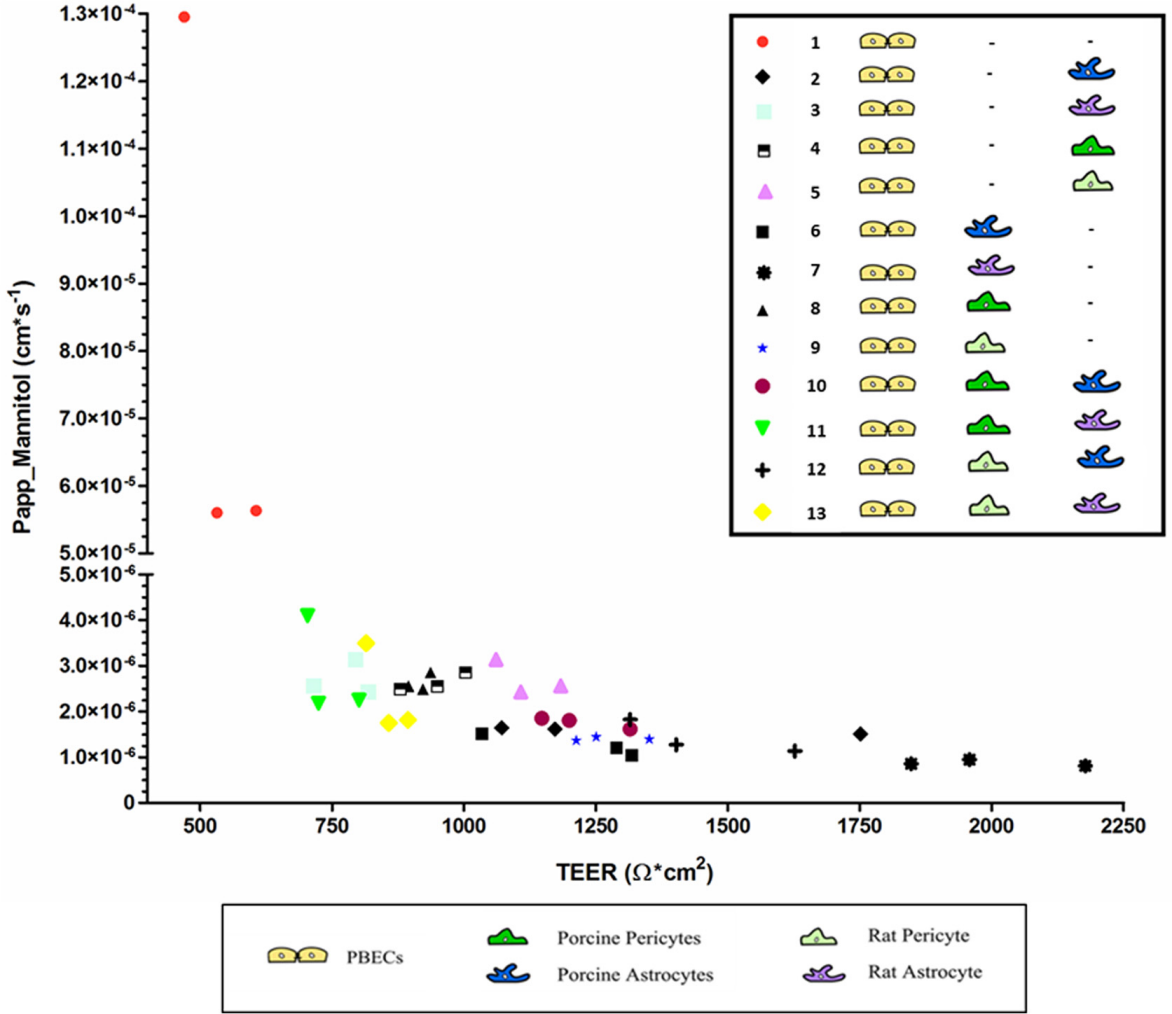
Mannitol permeability measurements on PBECs in thirteen co-culture combinations as a function of their TEER. TEER measured just before the permeability experiment was conducted. The Papp mannitol measured on n = 3 for each culture condition. Each point represents one hanging culture insert with PBECs.

The optimal properties of an *in vitro* BBB model are reflected in high expression of tight junction proteins that do not just lead to a high TEER but also low passive permeability of low-molecular substances like sodium fluorescein or mannitol from the luminal to the abluminal side of the *in vitro* BBB model. The permeability to mannitol was measured in cultures of PBECs in thirteen different culture conditions and plotted against TEER values measured on the same PBECs just before the permeability experiments was initiated (Fig 3).

Gaillard and de Boer found that there was an inverse relation between Papp and TEER [36], which also could be seen in the present study. All co- and triple cultures were in the range of 4.10–0.87 x 10^−6^ cm x s^-1^ and no significant differences were found between these culture conditions (Fig 3). The lowest permeability and hence highest integrity was found in PBECs cultured in co-contact with rat astrocytes, which had an average steady state mannitol permeability of 0.87 ± 0.04 x 10^−6^ cm x s^-1^ (Fig 3). This co-culture condition also had the highest TEER value before the permeability experiment was performed (1994 ± 79 Ω x cm^2^) (Fig 3). The highest permeability and hence lowest integrity was found in the monocultures, which had an average steady state mannitol permeability of 8.06 ± 2.4 x10^-5^ cm x s^-1^ (Fig 3). This was consistent with the TEER value; hence the monoculture had the lowest TEER value (535 ± 32 Ω x cm^2^) (Fig 3). The apparent mannitol permeability measured on the monoculture was significantly higher than on all the PBECs cultured in co- and triple cultures (P<0.0001).

Zhang et al. reported a comparable apparent mannitol permeability of 9.4 x 10^−5^ cm x s^-1^ for PBECs in monoculture with mean TEER of ~500 Ω x cm^2^ [22]. Franke et al. reported a permeability coefficient to mannitol in PBECs in monoculture as low as 1.8 x 10^−6^ cm x s^-1^, but with corresponding high peak TEER value of ~1500 Ω x cm^2^ [12]. These data correspond well with our permeability values of PBECs with a TEER of ~1500 Ω x cm^2^ (Fig 3). For non-contact co-cultures, mannitol permeability coefficient for PBECs in non-contact co-culture with rat C6 glioma cells was reported to be 2.31x10^-6^ cm x s^-1^ with corresponding TEER of 900 Ω x cm^2^ [24], and Papp to mannitol of PBECs in a non-contact co-culture with rat astrocytes at a range of 0.1–2.6 x 10^−5^ cm x s^-1^ and TEER values of ~800 Ω x cm^2^ [21]. No permeability coefficients can be found measured on PBECs in triple culture to compare with the results obtained in the present study.

All permeability values and corresponding TEER values reported in the literature on PBECs are in the same range as the ones found in the presents study (Fig 3) and, therefore, it seems that the inverse relationship is identical regardless of isolation procedures and to some extend also co-culture conditions.

At a certain level, Papp would not decrease any further despite of increasing TEER [36]. Previous studies have found that the apparent permeability of PBECs was relatively independent of TEER when TEER values were above 200–600 Ω x cm^2^ [12,21]. In the present study low and relatively steady permeability seems to be reached in between a TEER value of 606 Ω x cm^2^ and 704 Ω x cm^2^.

In conclusion PBECs in mono culture are significantly more permeable than PBECs cultured in co- and triple culture. No difference could be found between the permeability of PBECs cultured in co-culture and triple culture.

### mRNA expression in porcine brain endothelial cells

**Fig 4:**
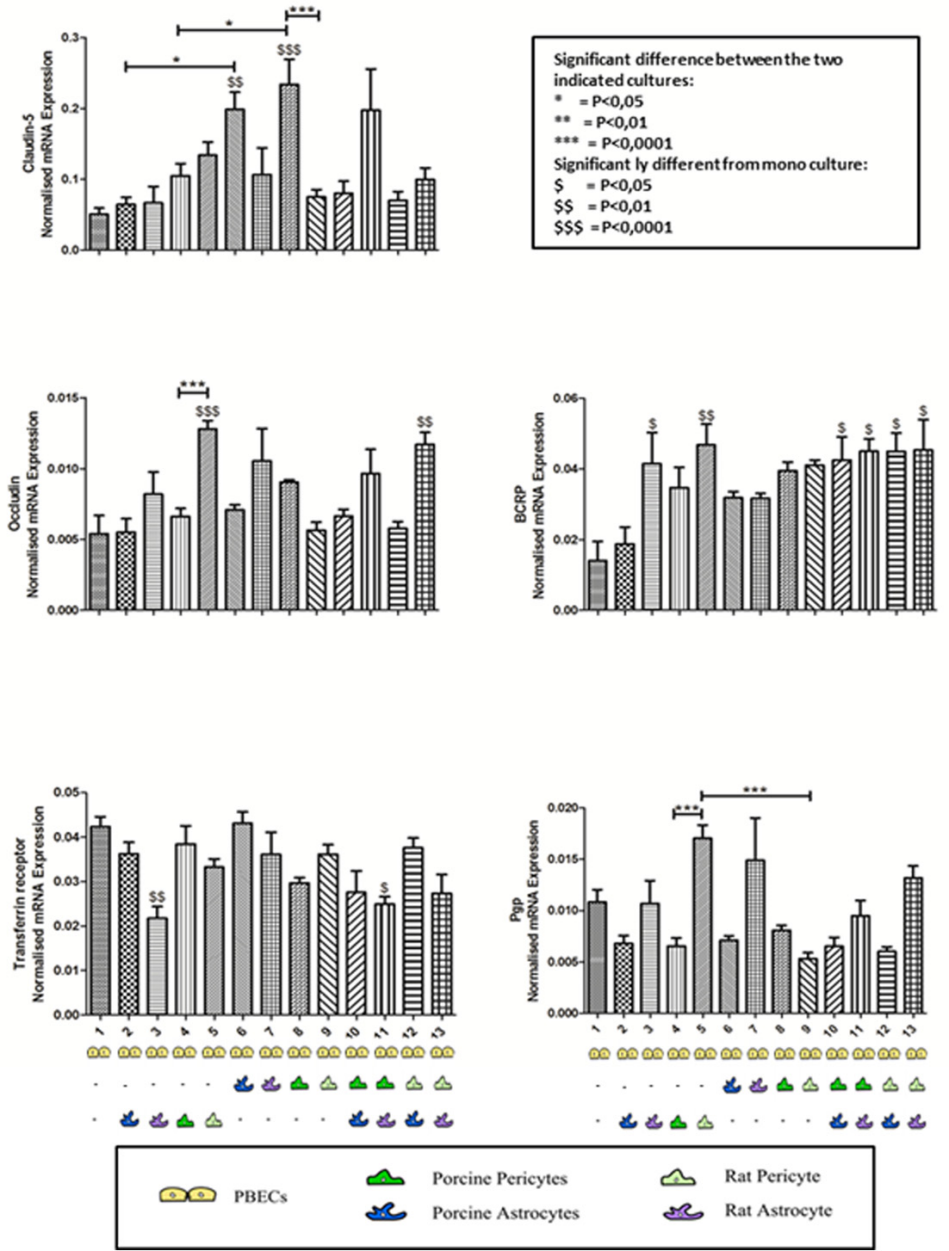
Gene Expression of claudin-5, Occludin, P-gp, BCRP and transferrin in PBECs. RT-qPCR performed on the PBECs from all thirteen different culture combinations. The relative gene expression of Claudin-5, Occludin, P-gp, BCRP and transferrin receptor-1 is shown for each culture combination. The results are given as relative expression normalized to beta-actin using the Pfaffl method (n = 3–6 replicates and one replicate consists of RNA from PBECs from 4–6 inserts).

The mRNA expression of claudin-5, occludin, transferrin receptor, P-gp and BCRP was confirmed by RT-qPCR (Fig 4). These molecules are all relevant for the BBB by means of being tight junction proteins (claudin-5, occludin), nutrient transporter (transferrin receptor) or drug/scavenger efflux transporters (P-gp, BCRP) and their expression indicates maintenance of important BBB features in the PBECs in culture. No significant differences were found in the relative mRNA expression of all the genes between PBECs grown in triple culture with porcine astrocytes and pericytes and PBECs cultured in triple culture with rat astrocytes and pericytes. Therefore, a triple culture model consisting entirely of porcine cells is preferred based on relative gene expression, when considering costs and ethics.

Expression of claudin-5 was significantly increased in PBECs when they were cultured in contact co-culture with either porcine astrocytes (P<0.01), porcine pericytes (P<0.0001) and triple culture with rat astrocytes and porcine pericytes (P<0.05) in comparison with mono cultures. The claudin-5 expression was significantly higher when PBECs were cultured in a contact co-culture with porcine pericytes (P<0.0001) compared to rat pericytes. This indicates that claudin-5 expression in PBECs depends on induction from porcine pericytes, which cannot be substituted by rat pericytes. Unfortunately, it also seems that when porcine astrocytes were included in the triple porcine culture, the inductive properties of the porcine pericyte were reversed, which was not seen when rat astrocytes were used instead for a triple culture with PBECs and porcine pericytes. The results also reveal that porcine astrocytes should be in contact with the PBECs for inducing the expression of claudin-5 due to a significant increase in expression (P<0.05) seen, when comparing with claudin-5 expression in PBECs in non-contact co-culture with porcine astrocytes. Malina et al., found similar results in PBECs in contact in co-culture with rat astrocytes where the protein expression of claudin-5 was significantly increased [35]. Porcine pericytes should also be cultured in contact rather than non-contact co-culture with the PBECs to induce a significant increase (P<0.05) in claudin-5 expression. Overall, we conclude that porcine pericytes are the most important cell type for increasing the expression of claudin-5 in PBECs when cultured in a contact co-culture.

Expression of occludin was only significantly increased by non-contact co-culture with rat pericytes (P<0.0001) and in triple culture with rat astrocytes and rat pericytes (P<0.05) when compared to monoculture. Furthermore, in non-contact co-culture rat pericytes significantly increased the occludin expression in PBECs compared to porcine pericytes in non-contact co-culture (P<0.0001). It should though be noted that there was no significant difference between the occludin expression in PBECs cultured in contact co-culture with either porcine or rat pericytes. It can, therefore, be concluded that occludin expression in PBECs is highly upregulated by co-culture with rat pericytes in a non-contact co-culture. The protein expression of occludin was increased although not significant when PBECs where co-cultured in contact with the astrocytes compared with PBECs in non-contact co-cultures with rat astrocytes [35]. However, in the present study PBECs co-cultured with rat astrocytes in non-contact co-culture have a higher relative mRNA expression of occludin than if they were co-cultured together in a contact co-culture.

P-gp expression in PBECs was not significantly increased by co-culture or triple culture with either porcine or rat astrocytes or pericytes. P-gp expression was increased significantly in PBECs by non-contact co-culture with rat pericytes when compared to non-contact co-culture with porcine pericytes (P<0.0001). The data also show that rat pericytes in contact co-culture with PBECs significantly decreased the P-gp expression compared to non-contact co-culture with rat pericytes (P<0.0001). It therefore seems that the highest expression of P-gp is achieved by culturing the PBECs in a non-contact co-culture with rat pericytes.

BCRP expression was significantly increased in PBECs in non-contact co-culture with rat astrocytes (P<0.01) and rat pericytes (P<0.05). Furthermore, BCRP expression was significantly increased in all four types of triple culture setups (P<0.05). There was no significant difference between co-culturing PBECs with rat or porcine astrocytes or pericytes. Although not significant the BCRP expression is upregulated in PBECs when they are cultured in co-culture and the expression is even higher when they are cultured in triple cultures. This up regulation seems to be independent of astrocyte and pericyte origin.

The transferrin receptor was present in PBECs in all culture setups. Transferrin receptor expression was only significantly decreased in PBECs by non-contact co-culture with rat astrocytes (P<0.01) and in triple culture with rat astrocytes and porcine pericytes (P<0.05). Therefore, rat astrocytes seem to decrease transferrin receptor expression, although this is not evident in triple culture with rat pericytes.

A tendency towards a higher expression of occludin (P<0.01), claudin-5, P-gp (P<0.05) and BCRP and lower expression of transferrin receptor was found when the PBECs were cultured in triple cultures with rat astrocytes and rat pericytes when compared to co-culture alone with either non-contact culture with rat astrocytes or contact co-culture with rat pericytes (Fig 4). It seems more complicated when comparing the mRNA expression in PBECs in the triple porcine culture with PBECs in either non-contact co-culture with porcine astrocytes or contact co-culture with porcine pericytes. The only significant difference was found in the expression of claudin-5 (P<0.01) which was increased in PBECs cultured in contact co-culture with porcine pericytes compared to PBECs in triple porcine cultures.

The differences in gene expression for all five molecules did not seem to be streamlined towards one type of culture setup. The differences seen could be due to differences in isolation and culture methods and this indicates that it is very important to have knowledge of exactly how the PBECs react in different culture setups to determine which conditions are the most optimal for different research purposes.

### Pericytes positively impact TEER, gene expression and permeability in co-culture and triple culture

That astrocytes impact TEER, gene expression and permeability of BECs is well known and has been widely investigated [5,22,32,33]. Fewer studies have been made on how pericytes impact BECs. Rat pericytes significantly increased TEER and decreased permeability of primary rat BECs in contact co-culture. Similar results were obtained in triple culture with rat pericytes cultured in contact with the BECs, when compared to rat BECs cultured either as monoculture or in co-culture with rat astrocytes [18].

In the present study, no significant difference was found between PBECs in co-culture with porcine/rat astrocytes and PBECs in a triple culture with porcine/rat astrocytes and porcine/rat pericytes (contact) either regarding gene expression, TEER or permeability.

The porcine pericytes significantly increase claudin-5 expression in contact culture with PBECs when compared to PBECs in non-contact co-culture with porcine astrocytes (P<0.01), and in triple culture with porcine astrocytes and porcine pericytes (contact) (P<0.01) (Fig 4). These observations indicate that the porcine pericytes are favorable to use in a contact-culture with PBECs with respect to introduction of claudin-5. Furthermore, rat pericytes are also important for obtaining a high expression of occludin and P-gp in PBECs when cultured in a non-contact co-culture (Fig 4).

## Conclusion

In the present study we successfully isolated PBECs, porcine astrocytes and pericytes together with rat astrocytes and pericytes. We constructed thirteen different mono-, co- and triple culture BBB models including a triple porcine in vitro BBB model. The porcine and rat cells express cell specific markers and high TEER values, and low permeability can be obtained with BECs of porcine origin in co- and triple cultures. We conclude that astrocytes and pericytes of either porcine or rat origin are equally good at inducing high TEER, low permeability and gene expression of the five investigated genes. Therefore an in vitro BBB model based on purely porcine cells can be preferred when taking expenses for animals, species differences and ethics into consideration. Due to the high cellular yield, the pure porcine BBB model has the advantage of being based on a single animal eliminating inter-individual differences.

## Supporting Information

S1 Fig In-vitro Blood-Brain Barrier model in different cell combinations. Thirteen different in-vitro BBB model combinations were constructed. 1) Mono culture of PBECs, 2) Non-contact co-culture of PBECs and porcine astrocytes, 3) Non-contact co-culture of PBECs rat astrocytes, 4) Non-contact co-culture of PBECs and porcine pericytes, 5) Non-contact co-culture of PBECs and rat pericytes, 6) Contact co-culture of PBECs and porcine astrocytes, 7) Contact co-culture of PBECs and rat astrocytes, 8) Contact co-culture of PBECs and porcine pericytes, 9) Contact co-culture of PBECs and rat pericytes, 10) Triple culture of PBECs, porcine astrocytes and porcine pericytes, 11) Triple culture of PBECs, rat astrocytes and porcine pericytes, 12) Triple culture of PBECs, porcine astrocytes and rat pericytes, 13) Triple co-culture of PBECs, rat astrocytes and rat pericytes.
(TIF)
