# Supplementary figures and images for: A Triple Culture Model of the Blood-Brain Barrier Using Porcine Brain Endothelial cells, Astrocytes and Pericytes
Source: PLoS One. 2015 Aug 4;10(8):e0134765. doi: 10.1371/journal.pone.0134765 (PMC4524625; doi:10.1371/journal.pone.0134765)

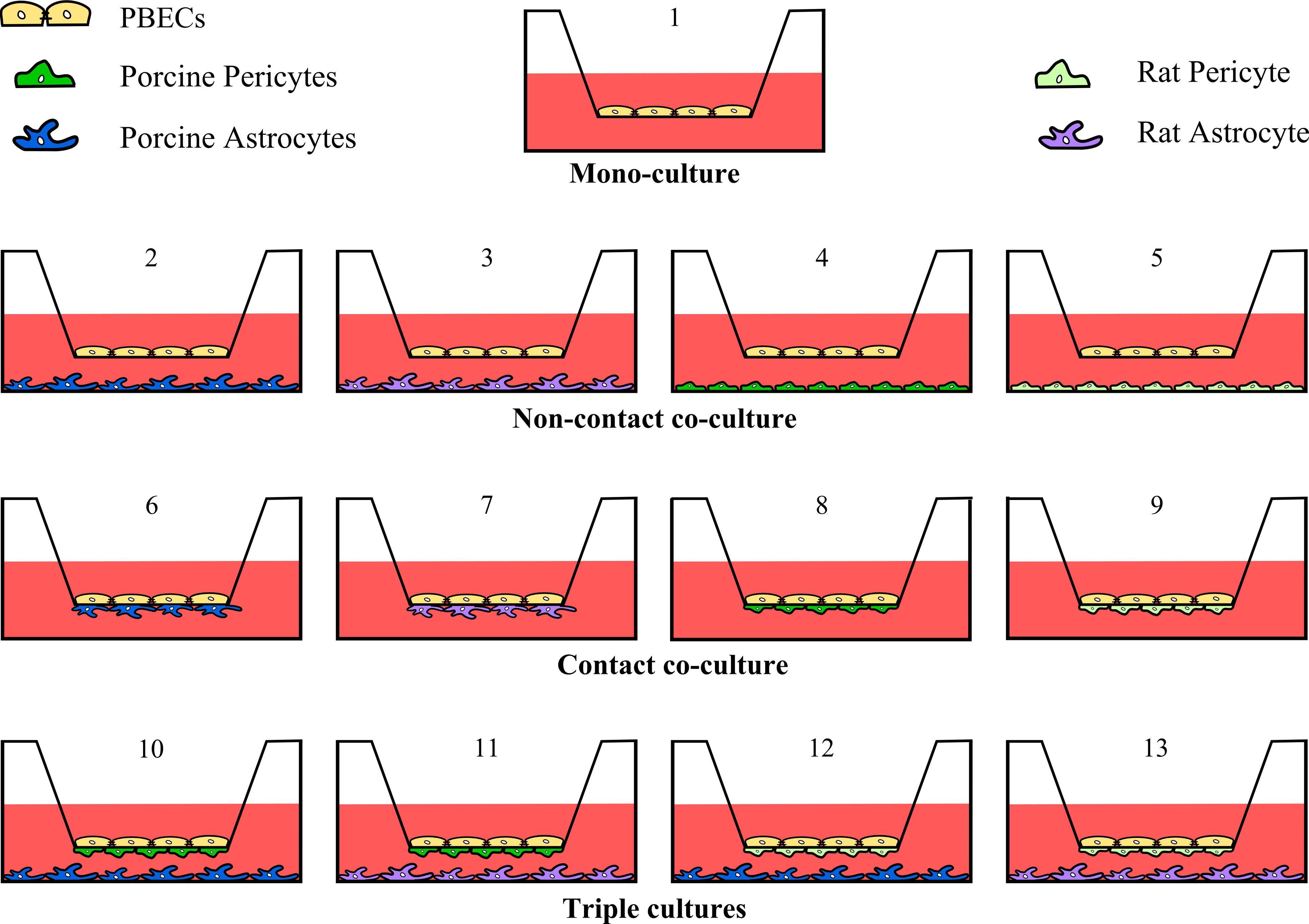

Supplement: S1 Fig — Thirteen different in-vitro BBB model combinations were constructed. 1) Mono culture of PBECs, 2) Non-contact co-culture of PBECs and porcine astrocytes, 3) Non-contact co-culture of PBECs rat astrocytes, 4) Non-contact co-culture of PBECs and porcine pericytes, 5) Non-contact co-culture of PBECs and rat pericytes, 6) Contact co-culture of PBECs and porcine astrocytes, 7) Contact co-culture of PBECs and rat astrocytes, 8) Contact co-culture of PBECs and porcine pericytes, 9) Contact co-culture of PBECs and rat pericytes, 10) Triple culture of PBECs, porcine astrocytes and porcine pericytes, 11) Triple culture of PBECs, rat astrocytes and porcine pericytes, 12) Triple culture of PBECs, porcine astrocytes and rat pericytes, 13) Triple co-culture of PBECs, rat astrocytes and rat pericytes. (TIF) [file pone.0134765.s001.tif]
